# Antioxidative and Anti-Inflammatory Effects of Water Extract of* Acrostichum aureum* Linn. against Ethanol-Induced Gastric Ulcer in Rats

**DOI:** 10.1155/2018/3585394

**Published:** 2018-12-12

**Authors:** Xue Wu, Qionghui Huang, Nan Xu, Jian Cai, Dandan Luo, Qian Zhang, Ziren Su, Changjun Gao, Yuhong Liu

**Affiliations:** ^1^Guangdong Provincial Key Laboratory of New Drug Development and Research of Chinese Medicine, Mathematical Engineering Academy of Chinese Medicine, Guangzhou University of Chinese Medicine, Guangzhou 510006, China; ^2^Guangdong Provincial Key Laboratory of Silviculture, Protection and Utilization, Guangzhou 510520, China; ^3^Guangdong Academy of Forestry, Guangzhou 510520, China

## Abstract

*Acrostichum aureum *Linn., a medicinal pteridophyte growing in mangrove forests and coastal regions of tropical and subtropical areas worldwide, has been proved to possess various biological effects. However, the protective effect of* Acrostichum aureum *Linn. against gastric ulcer still remains unidentified. Therefore, the gastroprotective effect of the water extract of* Acrostichum aureum *Linn. (WEAC) was investigated in ethanol-induced gastric injury model. According to our results, pretreatment with WEAC (100, 200, and 400 mg/kg) could dramatically decrease the ulcer areas and ameliorate the pathological damage induced by alcohol in rat's gastric tissues. In addition, WEAC administration prevented the stomach from oxidative damage via markedly increasing the levels of glutathione (GSH), superoxide dismutase (SOD), catalase (CAT), and decreasing the malondialdehyde (MDA). Besides, WEAC pretreatment alleviated inflammatory infiltration by reducing the secretion of proinflammatory cytokines including tumor necrosis factor-*α* (TNF-*α*), interleukin-1*β* (IL-1*β*), and interleukin-6 (IL-6) as well as decreasing the protein expressions of phosphorylation of I*κ*B*α* and p65. Taken together, WEAC exerted potential therapeutic efficacy for gastric ulceration, and this may be involved in the suppression of oxidative stress and inflammatory response.

## 1. Introduction

Mangrove plants are woody plants that grow in the tide tropical and subtropical zones over 121 countries. In the early stage of civilization, mangrove forests draw little attention because of their complicated geographical environment and difficulty to access. With the improvement of geographic information system, more and more mangrove forests were explored and confirmed to have multiple values by human being in recent decades. Many mangrove plants have been proved to possess a number of biological activities and extensively used to treat various diseases by the local inhabitants [1].* Acrostichum aureum *Linn., a mangrove fern belonging to the family Pteridaceae, mostly grows in mangrove forests and coastal regions of tropical and subtropical areas worldwide, especially in Southeast Asia, Central America, and Africa. Traditionally,* Acrostichum aureum *Linn. is broadly utilized for the treatment of diverse diseases. The rhizomes and leaves of* Acrostichum aureum *Linn. are widely used against worm infections, wounds, peptic ulcers, boils, and bleeding. And the roots of it are used to treat rheumatism, wounds, and boils [2]. Modern researches also confirm the anti-inflammatory [3] and antioxidantive [4, 5] activities of* Acrostichum aureum *Linn.

Gastrointestinal diseases are major health problems with high incidence and prevalence which affect millions of population worldwide [6]. As a common gastrointestinal disorder, gastric ulcer is usually caused by various noxious factors like* Helicobacter pylor*i, antiplatelet agents [7], smoking [8], alcohol [9], and chronic treatment with nonsteroidal anti-inflammatory drugs [10]. It is characterized by the classic signs of gastric mucosal ulceration, bleeding, and perforation, as well as an increased risk of other severe complications [11, 12]. Numerous studies have demonstrated that alcohol plays an important role in the damage of gastric mucosa and upper gastrointestinal bleeding [13, 14]. An unrestrained intake of alcohol may result in an imbalance between offensive and defensive factors of stomach and lead to gastric ulcer. Therefore, ethanol-stimulated gastric lesions model is commonly used to investigate the pathogenesis of gastric ulceration and evaluate the gastroprotective effect of drugs. The gastric ulcer caused by ethanol is related to high production of reactive oxygen species (ROS), which can trigger oxidative stress by inhibiting the effects of antioxidants including glutathione (GSH), superoxide dismutase (SOD), and catalase (CAT) [15]. ROS is also considered as a crucial offense factor in inflammation via increasing the formation of proinflammatory cytokines, such as tumor necrosis factor-*α* (TNF-*α*), interleukin-1*β* (IL-1*β*), and interleukin-6 (IL-6), [16] which exacerbate the inflammatory response.

Increasing evidence has shown that ethanol-induced gastric ulcer is closely linked to oxidative stress and inflammation, and the* Acrostichum aureum* Linn. has great antioxidative and anti-inflammatory effects. However, the antiulcer activity of* Acrostichum aureum* Linn. still remains unreported. Therefore, in this study, we prepared the water extract from the aerial parts of* Acrostichum aureum* Linn. (WEAC) and examined its gastroprotective effect against ethanol-stimulated gastric damage by measuring the oxidative indicators and proinflammatory cytokines in rats.

## 2. Materials and Methods

### 2.1. Drugs and Chemicals

Lansoprazole was provided by Tuobin Pharmaceutical Factory (Guangdong, Shantou, China). Sodium carboxymethylcellulose (CMC-Na) was supplied by Sigma-Aldrich Inc. (St Louis, USA). The kits for biochemical analysis of SOD, GSH, CAT, and MDA were obtained from Nanjing Jiancheng Bioengineering Institute (Nanjing, Jiangsu, China). ELISA kits for TNF-*α*, IL-1*β*, IL-6, and ROS measurement were purchased from Shanghai Enzyme-linked Biotechnology Co. Ltd (Shanghai, China). Primary antibodies against I*κ*B*α*, p-I*κ*B*α*, p65, p-p65, and *β*-actin were obtained from Affinity Biosciences Inc. (USA). Other reagents were of analytical grade.

### 2.2. Preparation of Extracts

*Acrostichum aureum *Linn. was collected from Nansha Wetland Park (Guangdong, Guangzhou, China) and further identified by Professor Xiao-ping Lai (Guangzhou University of Chinese Medicine, Guangzhou, China). Voucher samples (741113) were preserved for further reference in South China Botanical Garden. The aerial part of* Acrostichum aureum *Linn. was cut into about 10 cm segments and soaked in distill water (10% w/v) and then boiled (98°C) for 2 h. The extract was collected and the residue was boiled in water for second time. Collect the solution and filter the solution as it is warm. A Buchner funnel (120 mm) and qualitative filter paper (∅11cm) were used for filter. Subsequently, the filtered extract was evaporated under the temperature of 100°C and the concentrated solution was freeze-dried. The WEAC powder was stored in the refrigerator at 4°C for further experiments.

### 2.3. Preliminary Phytochemical Screening

The water extract was phytochemically evaluated to determine the presence of its major secondary metabolites such as flavonoids, reducing sugar, phenols, saponins, tannins, organic acids, alkaloids, anthraquinones, cardiac glycosides, and steroids using stander tests [17, 18].

### 2.4. Animals

Healthy male Wistar rats, weighting 180-220 g, were obtained from the Medical Experiment Animal Center of Guangzhou University of Chinese Medicine (SCXK (YUE) 2013-0034). They were housed at the condition of controlled temperature (22 ± 2°C) and humidity (65 ± 10%) with free access to food and water, under a 12-h light/dark cycles. All experimental protocols were performed in accordance with the Ethics Committee for the Welfare of Experimental Animals of Guangzhou University of Chinese Medicine.

### 2.5. Ethanol-Induced Gastric Lesions

Before any experience, rats were allowed to adapt to the laboratory environment for one week. Then, all rats were randomly assigned into six groups (*n*=8), including normal (0.5% CMC-Na), model (0.5% CMC-Na), LSZ (Positive control, 30 mg/kg lansoprazole dissolved in 0.5% CMC-Na), and WEAC (100, 200, and 400 mg/kg dissolved in 0.5% CMC-Na, respectively) groups. All rats were orally administered once daily for a period of 7 days. Prior to experimentation, all rats were fasted for 24 h with access only to clean water and accommodated in uniform mesh cages to prevent coprophagy. One hour after the final administration, animals except normal group were orally given with absolute ethanol (0.5 mL/100 g body weight) to establish acute gastric damage model, as previously mentioned method [19]. Blood samples from each rat were collected for serum biochemistry tests after 60 min, then animals were euthanized, and their stomachs were rapidly removed for further analysis.

### 2.6. Evaluation of Gastric Mucosal Damage

The stomach of each animal was opened along the greater curvature, douched with clean ice-cold saline to remove the gastric contents and blood clots. Subsequently, stomachs were stretched within two glass pane to photograph. The ulcer area was analyzed by ImageJ (1.47 v, National Institutes of Health, USA). The inhibition percentage was calculated according to the following formula:

(1) Ulcer  Area  (Model)  - Ulcer  Area  TreatedUlcer  Area  Model×100%.

### 2.7. Histopathological Analysis

After photographing, the stomach segments were fixed in 4% paraformaldehyde, dehydrated, and embedded in paraffin, and then stomach slices of 5 *μ*m thickness were papered for hematoxylin and eosin (H&E) staining. Histological evaluation was performed under a light microscope at 100× magnification according to the method previously reported [20]. The histopathological changes were evaluated by a pathologist who were blinded to this study according to the method with slight modifications [21]: (1) epithelial cell loss (score: 0–3), (2) hemorrhage (score: 0–4), and (3) inflammatory cell infiltration (score: 0–2). Summation of the three partial scores gave the total microscopic score.

### 2.8. Measurement of Oxidative Indicators

Tissues stored at −80°C were thawed and homogenized in ice-physiological saline, and 10% (w/v) homogenates were centrifuged at 4000 rpm and 4°C for 10 min to obtain supernatant for further analysis. The supernatant was collected and used to determinate SOD, GSH, CAT, and MDA activities according to manufacturer's instructions. The absorbance was analyzed by microplate reader.

### 2.9. Measurement of Proinflammatory Cytokines and ROS

Stomach tissues were homogenized in phosphate buffer saline (pH 7.2-7.4) to obtain 10% (w/v) homogenates. Then suspensions were centrifuged at 4000 rpm at 4°C for 10 min and the supernatants were collected for proinflammatory cytokines and ROS examination. Levels of TNF-*α*, IL-1*β*, IL-6, and ROS were measured by ELISA kits and the absorbance was read at 450 nm using microplate reader.

### 2.10. Western Blot

Total protein was extracted by RIPA Lysis Buffer with protease and phosphatase inhibitors following the manufacturer's instructions. Protein concentrations were determined using bicinchoninic acid assay (BCA) protein assay kit. Protein samples were separated on 10% SDS-PAGE (sodium dodecyl sulfate-polyacrylamide gel electrophoresis) and transferred to PVDF (polyvinyl difluoride) membranes. The membranes were blocked with 5% (w/v) skim milk in TBST, incubated with 1: 1000 dilution of primary antibodies (p-I*κ*B*α*, I*κ*B*α*, p-p65, p65, and *β*-actin) and the appropriate HRP-conjugated secondary antibody. Protein bands were detected using an ECL Advanced kit (Amersham Biosciences, Buckinghamshire, UK) and quantified by Quantity One 4.6.2 software. A *β*-actin antibody was used as the loading control.

### 2.11. Statistical Analysis

Statistical analysis was performed using a one-way analysis of variance (ANOVA) followed by an LSD test for multiple comparisons using Statistical Product and Service Solutions (SPSS) software (version 20.0). All data were exhibited as mean ± SD. *P*< 0.05 was allowed to forecast statistical significant.

## 3. Results

### 3.1. Phytochemical Study

Results of preliminary phytochemical screening on WEAC in Table 1 showed the presence of flavonoids, reducing sugar, phenols, saponins, tannins, organic acids, and steroids.

### 3.2. Effect of WEAC on the Gastric Mucosal Injury

**Figure 1:**
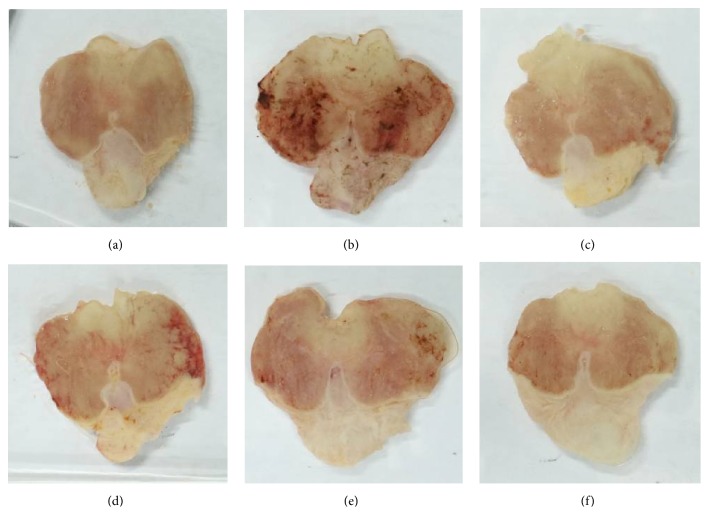
Effect of WEAC on the macroscopic appearance of gastric mucosa in rats (*n* = 8). (a) Normal group; (b) model group; (c) LSZ group (30 mg/kg); (d) WEAC (100 mg/kg) group; (e) WEAC (200 mg/kg) group; (f) WEAC (400 mg/kg) group.

**Figure 2:**
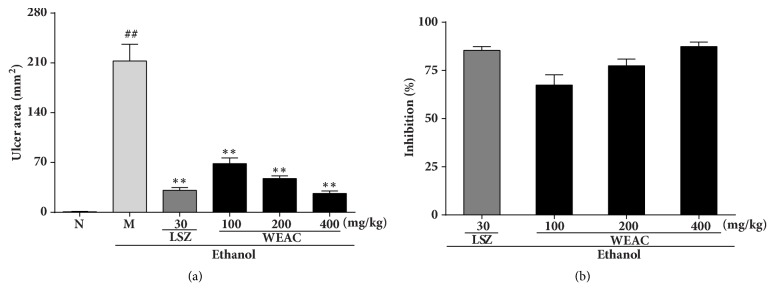
Effect of WEAC on the gastric ulcer area (mm^2^) and inhibition rate (%) in rats exposed to ethanol (*n* = 8). (a) Gastric ulcer area; (b) inhibition rate. Data are expressed as the mean ± SD. Compared with normal group: ^##^* p* < 0.01; compared with model group: **∗∗*** p < *0.01.

As revealed in Figures 1 and 2, after exposure to the alcohol, a severe (*p <* 0.01) hemorrhagic lesion with ulcer area of 212.69 ± 23.46 mm^2^ was displayed in model group. By contrast, a markedly decrease in the ulcer area was observed in LSZ (30 mg/kg) group with an ulcer area of 30.96 ± 3.94 mm^2^ (83.09% inhibition). Treatment with different doses of WEAC could remarkably attenuate the severe injury caused by ethanol in gastric mucosa. The best antiulcer effect was shown in 400 mg/kg WEAC group with the minimum ulcer area (26.57 ± 3.45 mm^2^) and the highest inhibition (87.74%).

### 3.3. Histological Evaluation

**Figure 3:**
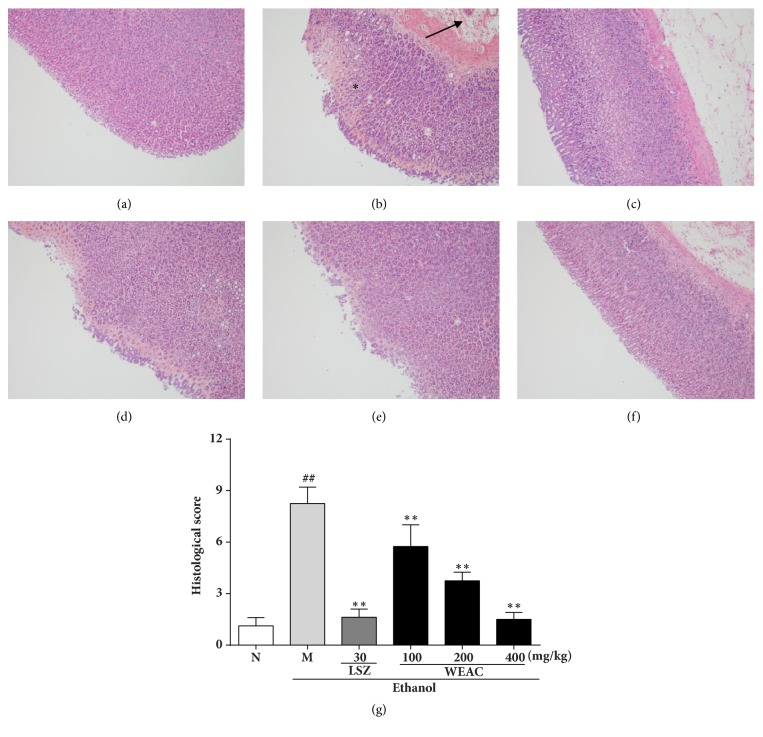
Effect of WEAC on histopathological lesions of gastric mucosa in rats exposed to ethanol (HE staining, 100×) (*n* = 8). (a) Normal group; (b) model group: the arrow indicates inflammatory cell infiltration and *∗* indicates high degree of hemorrhagic injury; (c) LSZ group (30 mg/kg); (d) WEAC (100 mg/kg) group; (e) WEAC (200 mg/kg) group; (f) WEAC (400 mg/kg) group; (g) histopathological score. Data are expressed as the mean ± SD. Compared with normal group: ^##^* p *< 0.01; compared with model group: **∗∗*** p < *0.01.

The results of the HE staining in Figure 3 showed that ethanol administration had manifested certain extensive gastric lesions accompanied with a severe hemorrhagic injury, epithelial cell loss, and inflammatory cell infiltration as compared to normal group. In contrast to the ethanol-treated group, pretreatment with LSZ alleviated the histological lesions by reducing hyperemia and epithelial cell loss in the stomach. Meanwhile, WEAC pretreatment also attenuated these alterations in a dose-dependent manner.

### 3.4. Effect of WEAC on ROS Production

**Figure 4:**
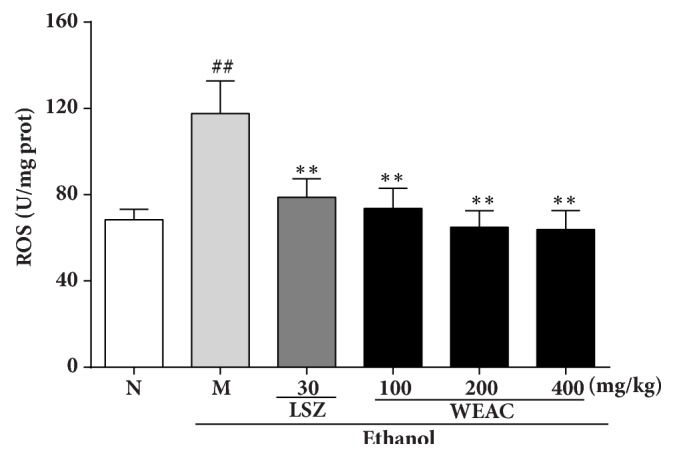
Effect of WEAC on the level of ROS in the stomach of rats exposed to ethanol (*n* = 8). Data are expressed as the mean ± SD. Compared with normal group: ^##^* p *< 0.01; compared with model group: **∗∗*** p < *0.01.

As shown in Figure 4, a significant increase in ROS level was found in model group when compared with the normal group. Specifically, ROS production of model group (117.61 ± 15.15 U/mg protein) significantly (*P <* 0.01) increased upon the normal group (68.44 ± 4.78 U/mg protein), which could be dose-dependently repressed by WEAC treatment. Results showed that the greater inhibitory activity for ROS production (63.80 ± 8.83 U/mg protein) was presented in 400 mg/kg WEAC group.

### 3.5. Effect of WEAC on SOD, GSH, CAT, and MDA Levels

**Figure 5:**
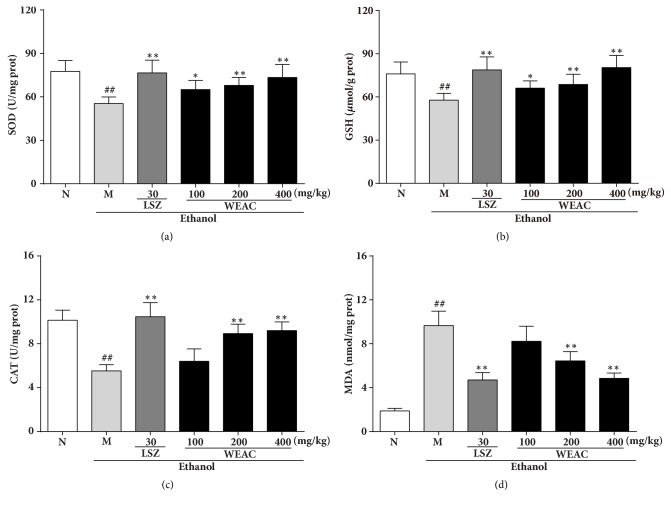
Effect of WEAC on the levels of (a) SOD, (b) GSH, (c) CAT, and (d) MDA in the stomach of rats exposed to ethanol (*n *= 8). Data are expressed as the mean ± SD. Compared with normal group: ^##^* p *< 0.01; compared with model group: *∗ p < *0.05; *∗∗ p < *0.01.

As shown in Figure 5. A markedly (*p <* 0.01) reduction of SOD (55.40 ± 4.53 U/mg protein), GSH (57.77 ± 4.74 *μ*mol/g protein), and CAT (5.53 ± 0.56 U/mg protein) was presented in ethanol-treated rats as compared with the normal group (SOD: 77.65 ± 7.39 U/mg protein; GSH: 76.04 ± 8.26 *μ*mol/g protein; CAT: 10.14 ± 0.92 U/mg protein), which could restore by LSZ and WEAC administration. Meanwhile, alcohol induced lipid peroxidation and significantly (p<0.01) increased the content of MDA in rats (9.66 ± 1.32 nmol/mg protein) as compared to the normal group (1.88 ± 0.23 nmol/mg protein), which was markedly reversed by WEAC and LSZ. The group with 400 mg/kg WEAC showed superior effect in elevating SOD (73.43 ± 8.92 U/mg protein), GSH (80.40 ± 8.41 *μ*mol/g protein), and CAT (9.20 ± 0.79 U/mg protein) levels and reducing MDA (4.85 ± 0.48 nmol/mg protein) level among all treated groups.

### 3.6. Effect of WEAC on Proinflammatory Cytokines Productions

**Figure 6:**
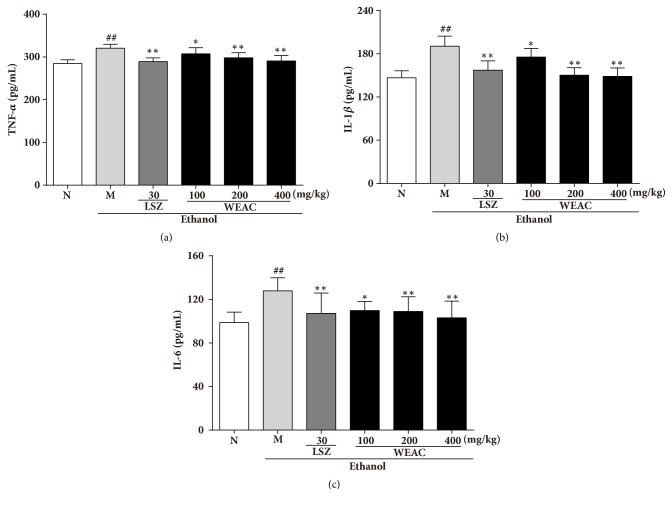
Effect of WEAC on the levels of (a) TNF-*α*, (b) IL-1*β*, and (c) IL-6 in the stomach of rats exposed to ethanol (*n *= 8). Data are expressed as the mean ± SD. Compared with normal group: ^##^* p *< 0.01; compared with model group: *∗ p < *0.05; **∗∗*** p < *0.01.

As showed in Figure 6, alcohol administration significantly (*P <* 0.01) increased TNF-*α* (320.53 ± 9.24 pg/ml), IL-1*β* (190.58 ± 13.97 pg/ml), and IL-6 (127.88 ± 12.06 pg/ml) levels as compared with the normal group (TNF-*α*: 284.47 ± 8.88 pg/ml; IL-1*β*: 146.63 ± 9.84 pg/ml; IL-6: 98.67 ± 9.62 pg/ml). However, rats pretreated with WEAC and LSZ could suppress the elevated TNF-*α*, IL-1*β*, and IL-6 levels in comparison with ethanol-treated group. Rats treated with 400 mg/kg WEAC showed the best effects on reducing TNF-*α* (290.52 ± 12.54 pg/ml), IL-1*β* (148.65 ± 11.75 pg/ml), and IL-6 (103.14 ± 15.30 pg/ml) levels.

### 3.7. Effect of WEAC on the NF-*κ*B Pathway

**Figure 7:**
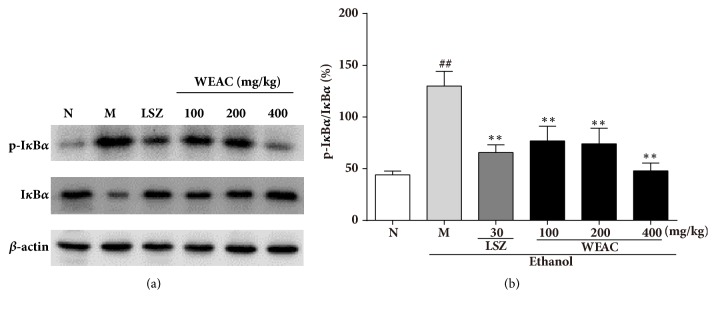
Effect of WEAC on the protein expressions of I*κ*B*α* and p-I*κ*B*α* in the stomach of rats exposed to ethanol (*n *= 3). (a) Representative Western blots. (b) Ratio of p- I*κ*B*α*/ I*κ*B*α*. Data are expressed as the mean ± SD. Compared with normal group: ^##^* p *< 0.01; compared with model group: **∗*** p < *0.05; **∗∗*** p < *0.01. *β*-actin was used as loading controls.

**Figure 8:**
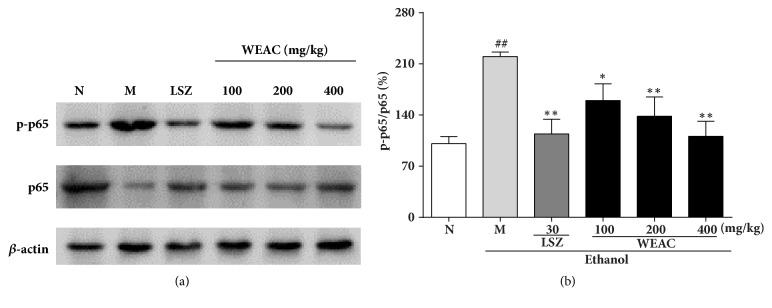
Effect of WEAC on the phosphorylation of NF-*κ*B p65 in the stomach of rats exposed to ethanol (*n *= 3). (a) Representative Western blot. (b) Ratio of p-p65/p65. Data are expressed as the mean ± SD. Compared with normal group: ^##^* p *< 0.01; compared with model group: **∗*** p < *0.05; **∗∗*** p < *0.01. *β*-actin was used as loading controls.

To uncover the underlying molecular mechanisms of the anti-inflammatory activity of WEAC on the NF-*κ*B pathway, we assayed the protein expressions of p65 and I*κ*B*α* by western blot. In Figures 7 and 8, the results revealed that the ratios of p-p65/p65 and p-I*κ*B*α*/I*κ*B*α* were dramatically decreased after treating with ethanol, which demonstrated that the phosphorylation of p65 and I*κ*B*α* was remarkably improved. However, this phosphorylation was notably attenuated by LSZ and WEAC pretreatment. Pretreatment with LSZ and WEAC decreased the ratios of p-p65/p65 and p-I*κ*B*α*/I*κ*B*α*, and the 400 mg/kg of WEAC was found to exert the best inhibition among all treated groups.

## 4. Discussion

Pteridophytes are a group of spore bearing, seed-less vascular plants which were appeared in the mid-Paleozoic era of the Silurian period and dominated landscapes during the Carboniferous and Permian [22]. With the introduction of ethnobotany, various parts of pteridophytes were found to be potential medicines for the treatment of different ailments.* Acrostichum aureum *Linn., a mangrove fern, has been proved to possess diverse pharmacological effects previously. However, the gastroprotective effect of the WEAC still remained uncertain. In this study, we first explained the prophylactic action of WEAC against the ethanol-stimulated gastric injury and provided mechanistic insight of its action. Results of this study demonstrated that pretreatment with WEAC dramatically alleviated gastric mucosal damage by reducing the oxidative stress and inflammatory response.

Oxidative stress has been linked to many pathological processes. ROS is often associated with the principle of oxidative stress. It is the by-product of aerobic metabolism which consists of superoxide anion (O_2_^*▪*–^), hydrogen peroxide (H_2_O_2_), and hydroxyl radicals (OH**·**). Evidences showed that ethanol administration significantly increases ROS generation [23] and causes DNA damage [24]. GSH, SOD, and CAT are vital endogenous antioxidants which protect biomembrane from oxidative damage by scavenging ROS. SOD, an intracellular enzymatic antioxidant, mainly converts harmful O_2_^*▪*–^ into less dangerous H_2_O_2_ and further metabolizes H_2_O_2_ into safe water [25]. In the mucosa, GSH is considered as an antioxidative barrier which protects the gastric mucosa against oxidative stress caused by free radicals and peroxides [26]. It also coordinates with some other antioxidant enzymes then alleviates oxidative damage. CAT can scavenge ROS by triggering the rapid conversion of peroxyl radical (H_2_O_2_^−^) into water and oxygen [27]. MDA is a metabolite for oxidative stress which is generated by unsaturated fatty acids through ROS-activated lipid peroxidation. Thus, MDA is deemed as the biomarker of lipid peroxidation and used to quantify and identify oxidative stress [28]. In this study, a marked increase of MDA level and decrease of SOD, GSH, and CAT levels were observed in rats after ethanol exposure. However, WEAC administration retrieved the depletion of GSH, SOD, and CAT and elevation of MDA level. These findings revealed that WEAC mainly suppressed the ethanol-induced gastric lesions by inhibiting oxidative stress.

A relationship between inflammation and the course of ethanol-stimulated gastric damage is widely recognized. Inflammation is a complex response of the host to tissue injury through an elevated secretion of proinflammatory cytokines, such as TNF-*α*, IL-1*β*, and IL-6 from immune cells [29]. TNF-*α* is one of the most representative proinflammatory cytokines which not only enhances the production of IL-1*β* and IL-6 but also induces the activation of NF-*κ*B through binding to the TNF-receptor [30, 31]. NF-*κ*B is a crucial transcription factor which has been widely deemed as the regular factor of immunity and inflammatory responses [32]. Normally, NF-*κ*B keeps in inactive state through binding of inhibitory protein I*κ*B*α* in uninduced cells. When encountering with multiple inducers such as TNF and ROS, the I*κ*B*α* protein becomes phosphorylated, ubiquitylated, and degraded [33]. Subsequently, NF-*κ*B is activated in response to phosphorylation of I*κ*B*α* and then is released from its dimer which contains Rel A (also known as p65) and NF-*κ*B [34]. Free NF-*κ*B translocates into nucleus and triggers the transcriptional activation of proinflammatory mediators [35]. In this study, WEAC pretreatment significantly reverted the levels of proinflammatory cytokines to their normal levels and inhibited the activation of NF-*κ*B pathway by inhibiting the phosphorylation of IkB*α* and p65. These results indicated that the mechanism of WEAC ameliorating gastric ulcer might associates with its anti-inflammatory effect through modulating the secretion of proinflammatory cytokines and the activation of NF-*κ*B signaling pathway.

## 5. Conclusion

Taken together, the findings of this research amply demonstrated that WEAC exerted appreciable gastroprotective effect against ethanol-induced gastric ulcer in rats. Administration with WEAC not only markedly increased the activities of CAT, SOD, and GSH, decreased the level of MDA, but also reduced the productions of TNF-*α*, IL-1*β*, and IL-6. Moreover, WEAC pretreatment suppressed the protein expressions of phosphorylation of IkB*α* and p65 to block the activation of NF-*κ*B. Therefore, it is reasonable to believe that the underlying mechanism of WEAC against gastric ulcer may be involved in the suppression of oxidative stress and inflammatory response. WEAC might be a promising agent to improve ethanol-induced gastric damage in rats.

## Figures and Tables

**Table 1:** Results of phytochemical screening of WEAC.

S. no	Tests	extract
1	Flavonoids	+
2	Reducing Sugar	+
3	Phenols	+
4	Saponins	+
5	Tannins	+
6	Organic acids	+
7	Steroids	+
8	Alkaloids	-
9	Anthraquinones	-
10	Cardiac glycosides	-

(+) present; (-) absent.

## Data Availability

The data used to support the findings of this study are available from the corresponding author upon request.

## References

[1] Rahim A. A., Rocca E., Steinmetz J., Kassim M. J., Adnan R., Sani Ibrahim M. (2007). Mangrove tannins and their flavanoid monomers as alternative steel corrosion inhibitors in acidic medium. *Corrosion Science*.

[2] Momtaz M. M. (2008). *Encyclopedia of Flora and Fauna of Bangladesh*.

[3] Hossain H. (2012). Antidiarrhoeal activity and total tannin content from the root of Acrostichum aureum (Linn). *International Journal of Pharmacy*.

[4] Xian Y., Li Y., Ip S., Lin Z., Lai X., Su Z. (2011). Anti-inflammatory effect of patchouli alcohol isolated from Pogostemonis Herba in LPS-stimulated RAW264.7 macrophages. *Experimental and Therapeutic Medicine*.

[5] Carneiro V. L., Fraiz F. C. (2003). Pharmacological studies of plants in the mangrove forest. *Revista Cubana De Estomatología*.

[6] Sumbul S., Ahmad M. A., Asif M., Akhtar M. (2011). Role of phenolic compounds in peptic ulcer: An overview. *Journal of Pharmacy and Bioallied Sciences*.

[7] Yeomans N. D., Hawkey C. J., Brailsford W., Næsdal J. (2009). Gastroduodenal toxicity of low-dose acetylsalicylic acid: A comparison with non-steroidal anti-inflammatory drugs. *Current Medical Research and Opinion*.

[8] Kurata J. H., Nogawa A. N. (1997). Meta-analysis of risk factors for peptic ulcer: nonsteroidal antiinflammatory drugs, *Helicobacter pylori*, and smoking. *Journal of Clinical Gastroenterology*.

[9] Chauhan A. K., Kang S. C. (2015). Therapeutic potential and mechanism of thymol action against ethanol-induced gastric mucosal injury in rat model. *Alcohol*.

[10] Takeuchi K. (2012). Pathogenesis of NSAID-induced gastric damage: importance of cyclooxygenase inhibition and gastric hypermotility. *World Journal of Gastroenterology*.

[11] Oates P. J., Hakkinen J. P. (1988). Studies on the mechanism of ethanol-induced gastric damage in rats. *Gastroenterology*.

[12] Sid B., Verrax J., Calderon P. B. (2013). Role of oxidative stress in the pathogenesis of alcohol-induced liver disease. *Free Radical Research*.

[13] Franke A., Teyssen S., Singer M. V. (2006). Alcohol-related diseases of the esophagus and stomach. *Digestive Diseases*.

[14] Ma L., Liu J. (2014). The protective activity of Conyza blinii saponin against acute gastric ulcer induced by ethanol. *Journal of Ethnopharmacology*.

[15] Rezaie A., Parker R. D., Abdollahi M. (2007). Oxidative stress and pathogenesis of inflammatory bowel disease: an epiphenomenon or the cause?. *Digestive Diseases and Sciences*.

[16] Nian M., Lee P., Khaper N., Liu P. (2004). Inflammatory cytokines and postmyocardial infarction remodeling. *Circulation Research*.

[17] Harborne J. B. (1998). Tony Swain and phytochemical methods. *Phytochemistry*.

[18] Derebe D., Abdulwuhab M., Wubetu M., Mohammed F. (2018). Investigation of the Antidiarrheal and Antimicrobial Activities of 80% Methanolic Leaf Extract of Discopodium Penninervum (Hochst.). *Evidence-Based Complementary and Alternative Medicine*.

[19] Liu Y., Liang J., Wu J. (2017). Transformation of patchouli alcohol to *β*-patchoulene by gastric juice: *β*-patchoulene is more effective in preventing ethanol-induced gastric injury. *Scientific Reports*.

[20] Laine L., Weinstein W. M. (1988). Histology of alcoholic hemorrhagic ‘gastritis’: a prospective evaluation. *Gastroenterology*.

[21] Yang Y., Yin B., Lv L. (2017). Gastroprotective effect of aucubin against ethanol-induced gastric mucosal injury in mice. *Life Sciences*.

[22] Niklas K. J., Tiffney B. H., Knoll A. H. (1983). Patterns in vascular land plant diversification. *Nature*.

[23] Bilici D., Süleyman H., Banoglu Z. N. (2002). Melatonin prevents ethanol-induced gastric mucosal damage possibly due to its antioxidant effect. *Digestive Diseases and Sciences*.

[24] Cross C. E., Halliwell B., Borish E. T. (1987). Oxygen radicals and human disease. *Annals of Internal Medicine*.

[25] Halabi M. F., Shakir R. M., Bardi D. A. (2014). Gastroprotective Activity of Ethyl-4-[(3,5-di-tert-butyl-2-hydroxybenzylidene) Amino]benzoate against Ethanol-Induced Gastric Mucosal Ulcer in Rats. *PLoS ONE*.

[26] Brzozowski T., Konturek P. C., Konturek S. J. (2004). Exogenous and endogenous ghrelin in gastroprotection against stress-induced gastric damage. *Regulatory Peptides*.

[27] Wong J.-Y., Abdulla M. A., Raman J. (2013). Gastroprotective Effects of Lion’s Mane Mushroom *Hericium erinaceus* (Bull.:Fr.) Pers. (Aphyllophoromycetideae) Extract against Ethanol-Induced Ulcer in Rats. *Evidence-Based Complementary and Alternative Medicine*.

[28] Ibrahim I. A. A., Abdulla M. A., Hajrezaie M. (2016). The gastroprotective effects of hydroalcoholic extract of Monolluma quadrangula against ethanol-induced gastric mucosal injuries in Sprague Dawley rats. *Drug Design, Development and Therapy*.

[29] Nathan C. (2002). Points of control in inflammation. *Nature*.

[30] Zhou Y.-H., Yu J.-P., Liu Y.-F. (2006). Effects of Ginkgo biloba extract on inflammatory mediators (SOD, MDA, TNF-alpha, NF-kappa Bp65, IL-6) in TNBS-induced colitis in rats. *Mediators of Inflammation*.

[31] Bradley J. R. (2008). TNF-mediated inflammatory disease. *The Journal of Pathology*.

[32] El Eter E., Hagar H. H., Al-Tuwaijiri A., Arafa M. (2005). Nuclear factor-*κ*B inhibition by pyrrolidinedithiocarbamate attenuates gastric ischemia-reperfusion injury in rats. *Canadian Journal of Physiology and Pharmacology*.

[33] Tak P. P., Firestein G. S. (2001). NF-*κ*B: a key role in inflammatory diseases. *The Journal of Clinical Investigation*.

[34] Brown M. A., Jones W. K. (2004). NF-*κ*B action in sepsis: the innate immune system and the heart. *Frontiers in Bioscience*.

[35] Li W., Wang X., Zhang H. (2016). Anti-ulcerogenic effect of cavidine against ethanol-induced acute gastric ulcer in mice and possible underlying mechanism. *International Immunopharmacology*.

